# Effects of a 12-Week Hatha Yoga Intervention on Metabolic Risk and Quality of Life in Hong Kong Chinese Adults with and without Metabolic Syndrome

**DOI:** 10.1371/journal.pone.0130731

**Published:** 2015-06-25

**Authors:** Caren Lau, Ruby Yu, Jean Woo

**Affiliations:** Department of Medicine and Therapeutics, The Chinese University of Hong Kong, Hong Kong, China; Weill Cornell Medical College Qatar, QATAR

## Abstract

**Objective:**

To determine the efficacy of a 12-week Hatha yoga intervention to improve metabolic risk profiles and health-related quality of life (HRQoL) in Chinese adults with and without metabolic syndrome (MetS).

**Methods:**

We conducted a controlled trial within an university-affiliated hospital. 173 Chinese men and women aged 18 or above were assigned to either the yoga intervention group (n = 87) or the control group (n = 86). Primary outcomes included 12-week change in metabolic risk factors and MetS z score. Secondary outcome was HRQoL (Medical Outcomes Short Form Survey at 12 weeks).

**Results:**

The mean age of participants was 52.0 (SD 7.4, range 31-71) years. Analysis involving the entire study population revealed that the yoga group achieved greater decline in waist circumference (p<0.001), fasting glucose (p<0.01), triglycerides (p<0.05), and MetS z score (p<0.01). Yoga training also improved general health perceptions (p<0.01), physical component score (p<0.01), and social functioning (p<0.01) domains score of HRQoL. However, no significant differences between groups were observed in the mean change of systolic/diastolic blood pressures or high-density lipid protein cholesterol (all p>0.05). There were no significant differences in the intervention effects on waist circumference and MetS z score between the MetS subgroups (both p>0.05).

**Conclusion:**

A 12-week Hatha yoga intervention improves metabolic risk profiles and HRQoL in Chinese adults with and without MetS.

**Trial Registration:**

Australian New Zealand Clinical Trials Registry ACTRN12613000816752

## Introduction

The metabolic syndrome (MetS) has been described as a pandemic, with rapidly increasing prevalence worldwide over the past 20 years.[1] While there is evidence that the prevalence of MetS is decreasing in the US, [2] its prevalence is still increasing in Asian countries.[3] Given the increasing prevalence and the associated premature mortality, disability, and health and social economic costs, [4, 5] management of MetS is of importance to public health. Previous studies have shown that physical activity plays an integral role in preventing MetS.[6] Many individuals, however, are unable or unwilling to practice in conventional types of physical activity, such as aerobic exercises and resistance training, which have been shown to be beneficial in the prevention of MetS.[6]

Originated in India, yoga has become increasingly popular in western countries.[7] Of the various branches of yoga (such as Hindu, Hatha, Raja, and Mantra), Hatha yoga is perhaps the most widely practiced, which consists of elements of physical postures, conscious breathing, and mediation.[8] With no appreciable side effects, yoga appears safe and easy to learn, and does not require any complicated or expensive equipment or specific training venue, and thus could be suggested as an alternative form of physical activity. Although the overall level of intensity is thought to be low,[9] some yoga postures may achieve the recommended level of intensity for cardiovascular fitness.[10, 11] A growing number of research studies have shown that Hatha yoga can lead to improvements in muscle strength and flexibility,[12] maximal oxygen uptake (VO_2max_),[13] and may enhance psychological well-being.[14] Furthermore, its effects on indices of insulin resistance (e.g., fasting glucose) are robust and promising.[15] However, its positive effects on other metabolic risk factors (i.e. waist circumference, lipids, and blood pressure) remain inconclusive,[16] and most of the studies have methodological or other limitations such as the absence of comparisons groups and low power due to small sample sizes. In sum, this line of research is still in its infancy, and there have been relative few studies in Chinese populations, and the results regarding therapeutics effects of yoga on MetS as a whole are sparse.

To fill these knowledge gaps, we developed a 12-week Hatha yoga intervention in the community. We evaluated the intervention in a two-arm 12-week, prospective, non-blinded controlled trial enrolling Hong Kong Chinese with and without MetS. We hypothesized subjects participated in the yoga group would have improved metabolic risk profile than those in the control group. Secondary outcome was health-related quality of life (HRQoL) at 12-week.

## Methods

### Subjects

173 Chinese men and women aged 18 or above were recruited for the study between May 2010 and January 2011. Recruitment was done by newspaper advertising, by placing notices in community centers, and by Internet publicity (including emails, advertisement, discussion forums, and website). Subjects were volunteers, and the aim was to recruit a stratified sample so that similar proportions of male and female and of individuals with and without MetS were obtained. An enrolment form was used for the screening purposes. Chinese individuals aged 18 and older, able to communicate in Cantonese, and physically and mentally capable of practicing yoga safely were included. Those with a history of cognitive impairment, present severe illness, regularly participation (>1/week) of yoga, Qigong or have been on medication for last three months, were concurrently undergoing non-pharmacological treatment of MetS or participation in other research studies were excluded. A sample size of 46 participants per group was determined which would allow us to detect a difference of 0.08 g/dl in salivary cortisol level between the yoga and the control groups [17] using independent two sample mean test, with alpha = 0.05, power = 0.8, and an estimated attrition rate of 22%. However, the effects of the yoga intervention on salivary cortisol levels were not examined in this analysis.

### Procedure

**Fig 1:**
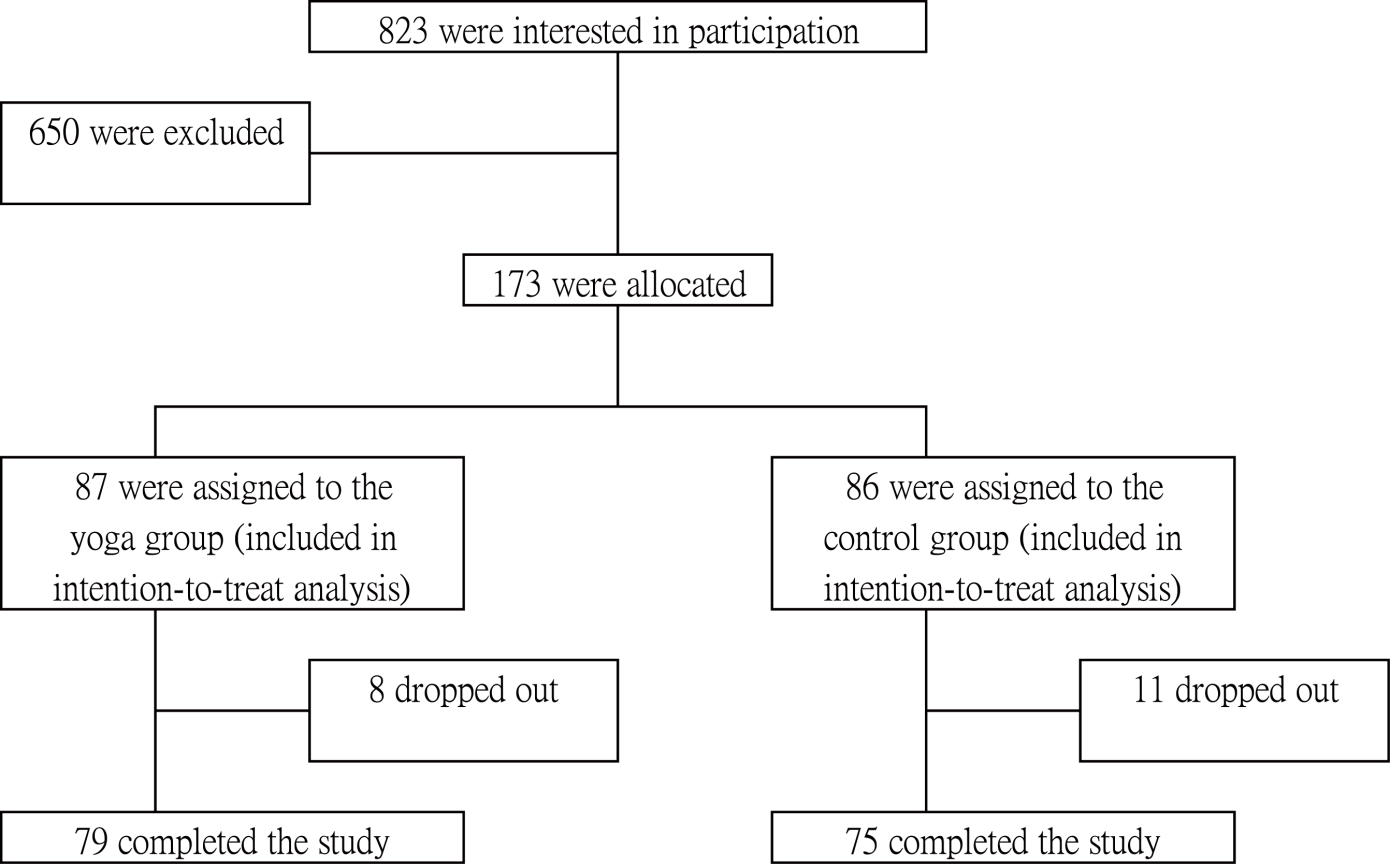
Recruitment of subjects.

The study had adopted a prospective two-arm non-blinded controlled design. Each subject was individually assessed and they were grouped as 87 for yoga and 86 for control groups (Fig 1). To ensure the proportions of male/female and of individuals with/without MetS in the yoga and control groups would be similar and comparable for subgroup analysis, quota sampling was adopted, with gender and presence of MetS used as quota controls. Individualss who met the inclusion criteria were invited to either the yoga or the control groups according to quota controls. They were not allowed to select their groups. To ensure acceptance of the protocol, waitlist control groups were offered a 12-hour yoga training upon completion of the study. Demographics, medication use, lifestyle factors, and HRQoL measures (described below) was taken before and after the 12-week protocol Baseline assessments were performed between July 2010 and January 2012. Follow-up assessments were performed between October 2010 and May 2012. The study was conducted within an university-affiliated hospital (Prince of Wales Hospital, Shatin, New Territories, Hong Kong). All eligible prospective participants participated voluntarily and their written informed consent was obtained prior to the study. The study was conducted as per the tenets of the Declaration of Helsinki with approval from the Joint Chinese University of Hong Kong-New Territories East Cluster Clinical Research Ethics Committee (Registration number: CRE-2010.115; Date of Approval: 27 April 2010). After obtaining the ethics approval, a feasibility trial was carried out (as a PhD project). Preliminary data were analysed and the results were encouraging. Therefore, a proper trial was being planned and the protocol has been retrospectively registered in the Australian New Zealand Clinical Trials Registry (Registration number: ACTRN12613000816752). The authors confirm that all on-going and related trials for this intervention are registered.

### Yoga training

The yoga group participants were invited to attend a group yoga training consisting of 12 weekly 60-minute sessions. The frequency of sessions was chosen partly for practical reasons because about half (53.2%) of the participants engaged in full time jobs and they could only attend the class once per week. Previous studies have also demonstrated that 12 once-weekly yoga classes are effective for improving social functioning and physical activity levels in women.[18, 19] The yoga groups were conducted by an Experienced Registered Yoga Teacher (E-RYT) (Yoga Alliance) with more than four years of Hatha yoga instructing experience. Throughout the 12 training sessions, participants were arranged in groups of seven to ten, and were taught the breathing technique and 57 yogic poses commonly taught in community fitness centres including 1) Standing poses: Chair Pose (*Utkatasana*) and its variation (i.e., Chair with Torso Twist), Extended Hand-Toe Pose (*Utthita Hasta Padangusthasana*), Extended Side Angle Pose (*Utthita Parsvakonasana*), Half Moon (*Ardha Chandrasana*), Modified Half Moon, Intense Side Stretch (*Parsvottanasana*), King of the Dancers Pose (*Natarajasana*), Lunge, Mountain Pose (*Tadasana*), Revolved Side Angle Pose (*Parivrtta Parshvakonasana*), Revolved Lunge, Revolved Triangle Pose (*Parivrtta Trikoasana*), Squat-Sitting-Down Pose (*Malasana*), Standing Forward Bend (*Uttanasana*), Tree Pose (*Vrkshasana*), Triangle Pose (*Trikonasana*), Warrior I (*Virabhadrasana I*), Warrior II (*Virabhadrasana II*), Warrior III (*Virabhadrasana III*), Wide-Stance Forward Bend (*Prasarita Padottansana*) and its variation (i.e., Wide-Stance Forward Bend with Torso Twist), 2) Sitting poses: Boat Pose (*Navasana*), Bound Angle Pose (*Baddha Konasana*), Cow-Face Pose (*Gomukhasana*), Half Load of the Fishes Pose (*Ardha Matsyendrasana*), Head-to-Knee Pose (*Janu Shirshasana*), Marichi I (*Marichyasana I*), Pigeon Pose (*Raja Kapotasana*), Revolved Head-to-Knee Pose (*Parivrtta Janu Shirshanasana*), Seated Forward Bend (*Paschimottanasana*), Seated Wide-Angle Pose (*Upavistha Konasana*), 3) Kneeling poses: Camel Pose (*Ushtrasana*), Child’s Pose (*Balasana*), Gate Pose (*Parighasana*), One-Legged Royal Pigeon and Folded Forward (*Raja Kapotasana*), 4) Suprine: Apana Pose (*Apanasana*), Belly Twist (*Jathara Partivartanasana*), Bridge Pose (*Setu Bandhasana*) and its variation (i.e., Bridge with One Leg Lift), Corpse Pose (*Shavasana*), Preparation exercise for Plow Pose, Plow Pose (*Halasana*), Reclining Bound Angle Pose (*Supta Baddha Konasana*), Side-Reclining Leg Lift (*Anantasana*), Supported Shoulder Stand (*Salamba Sarvangasana*), 5) Prone poses: Bow (*Dhanurasana*), Cobra (*Bhujangasana*), Locust (*Shalabhasana*), Upward Facing Dog (*Urdhva Mukha Shvanasana*), and 6) Arm support poses: Cat Cow (*Durga Go*) and its variation (i.e., Cat Cow with One Leg Lift), Four-Footed Table Top Pose (*Chatus Pada Pitham*), Plank (*Utthita Hasta Padangusthasana*), Table Top Exercise, Side Plank (*Vasishthasana*), Upward Plank (*Purvottanasna*). Participants were also encouraged to practice yoga at home between classes (with handouts of yogic poses), and self-practice log sheets were used. Apart from the yoga program, the yoga participants were advised to maintain their routine activities and not to begin other exercise or mind-body program during the course of the study.

### Control group

The control group participants were requested to maintain their routine activities and not to begin any exercise, yoga or mind-body program during the course of the study. To ensure adherence of the protocol, the control group participants also received the yoga program after the end of the study period.

### Primary outcomes

#### Metabolic risk factors

With the participant seated, a 21-gauge multisample needle with holder was used to perform a minimally traumatic venipuncture before noon after 12 hours of fasting. Fasting 6 mL blood samples were used for analysis of triglycerides, high-density lipoprotein cholesterol (HDL-C), and fasting glucose. Blood measurements were conducted at the Prince of Wales Hospital Chemical Pathology Laboratory (accredited by the National Association of Testing Authorities). All blood measurements were analyzed in a blinded manner.

Systolic and diastolic blood pressures (SBP, DBP) were measured using the automatic blood pressure device (HEM7011, Omron, Japan). According to American Heart Association (AHA) blood pressure measurement recommendations,[20] the participant was requested to remove all clothing that covered the location of cuff placement. The participant was comfortably and quietly seated for three to five minutes with the legs uncrossed, and the back and arm supported, such that the middle of the cuff on the upper arm is at the level of the right atrium. Three readings were taken in succession, separated by at least one minute. The first is typically the highest, and the average of the second and third systolic and diastolic pressure readings was used in the analyses. Waist circumference was measured at the nearest 0.1 cm at the shortest point below the lower rib margin and the iliac crest.

#### MetS z score

A continuous MetS risk score (MetS z score) was used to assess the metabolic risk, which was calculated by summing the standardized values for waist circumference, triglycerides, HDL-C, blood pressure, and fasting glucose. Gender-specific MetS z scores were used to account for variations in NTEC-ATP III criteria for men and women. Each variable was standardized by subtracting the sample mean from the individual mean and dividing by standard deviation. Both baseline and post intervention z scores were calculated with same transformation. It has been suggested that continuous score would be more sensitive to both small and large changes and less susceptible to errors than dichotomous approaches.[21] Recent studies have also chosen to adopt the continuous score instead of categorical data to analyze the association between physical exercise and MetS and its components.[22, 23]

### Secondary outcome

#### Health-related quality of life (HRQoL)

HRQoL was assessed with the Medical Outcomes Study (MOS) 36-item Short-Form Health Survey (SF-36),[24] which is a generic assessment of HRQoL, consists of 36 items grounded under eleven questions. SF-36 has the eight scales measuring physical functioning, role limitations due to physical problems, bodily pain, general health perceptions, vitality, social functioning, role limitations due to emotional problems, and general mental health. The physical health component score and mental health component score are two summary scores which provide overall assessment of quality of life related to physical and mental health, respectively. The internal consistency of the SF-36 ranged from 0.63 to 0.96 and the test-retest reliability ranged from 0.60 to 0.81.[24] The Chinese version of the SF-36 was employed in the present study. It has been validated in Chinese adults in Hong Kong.[25] A norm reference score for Hong Kong Chinese adults has also been reported and compared with US population norms.[26]

### Covariates

Age was treated as a continuous variable. Socio-demographic variables included marital status (single, married, widowed), education level (No education, primary, secondary, tertiary) and employment status (full-time, part-time, unemployed, housewives, retired). Medication use was also obtained. Lifestyle factors included smoking, alcohol intake, and physical activity. Smoking status was divided into three categories (current, quitted, never). Alcohol intake was divided into four categories (quitted, never, sometimes, always). Physical activity was measured with the short (seven day) form of the International Physical Activity Questionnaire (IPAQ).[27] The frequency and duration of sitting, walking, moderate-intensity, and vigorous-intensity physical activity across the domains of leisure-time, domestic activities, work-related activities and transportation were assessed. Height and body weight were measured in light clothing without shoes, using a digital standing scale. Body mass index (BMI) was calculated as kg/m^2^.

### Data processing and analysis

We analysed the outcome variables in the intention-to-treat (ITT) population, consisting of participants who completed the study protocol and those completed the baseline assessment but dropped out from the study afterward. We used carry-forward imputation to estimate the missing follow-up data in ITT population. Continuous and categorical variables were summarised as mean (SD), or by counts and percentages. We checked the homogeneity of the yoga and control groups with independent *t*-test and Mann-Whitney U test for continuous variables and Chi-square (χ^2^) test for categorical variables. Relationships between baseline characteristics and the outcome variables were determined with correlations. By incorporating findings from homogeneity check and correlations, possible covariates were identified (i.e., DBP, role emotional domain score, mental health component score). We tested the mean difference of each outcome variable between the yoga and control groups with analysis of variance (ANOVA) or analysis of covariance (ANCOVA) when possible covariates were identified. To assess the magnitude and direction of the effect of the yoga intervention relative to the control condition for each outcome variable, effect sizes were computed, where the value of partial eta squared (η^2^) represents very small (< 0.01), small (0.01–0.05), medium (0.06–0.13), and large (≥ 0.14). Interaction between yoga/control and exposure to MetS (MetS/nonMetS) was tested by addition of cross-product terms to the multivariate models. The analyses were repeated for the subgroups with and without MetS. Covariates adjusted included DBP (among MetS participants) and role emotional/mental health component score domains score (among non-MetS participants). All statistical tests were two-tailed and the acceptance level of statistical significance (p-value) in overall analysis was 0.05 or less. All statistical analyses were carried out using Windows-based Statistical Package for the Social Sciences version 16.0 software (SPSS, Chicago, IL, USA).

## Results

### Flow of participants and baseline characteristics

A total of 823 individuals were screened for eligibility. Sixty-six individuals were found to be not eligible due to physical health status. Thirty-nine prospective participants declined to be enrolled (reasons including failed to make themselves available for attending the assessments / most of the yoga classes etc.). Thirty-four prospective participants could not be contacted. Among the 511 prospective participants, 173 Chinese men (n = 64) and women (n = 109) were recruited and assigned to either the yoga group (n = 87) or the control group (n = 86), of which 19 dropped out from the study. Except for the general mental health domain score (p < 0.05), there were no significant differences in baseline characteristics between those retained and those lost to follow-up (data not shown). The mean age of the study population was 51.98 ± 7.46 (range 31–71) years. Most of the subjects were married (80.5–82.6%) and had secondary education (93.0–94.2%), and about half engaged in full-time jobs (52.3–55.2%). The mean physical activity levels were 1701.91–1796.33 MET-minutes/week. There were no significant differences in age, gender, marital status, educational level, occupation, medication use, smoking, alcohol intake, physical activity, body weight, MetS risk factors, and other HRQoL domains measure between the two groups at baseline (all p > 0.05) except for BMI (yoga: 24.44 ± 3.84 kg/m^2^; control 25.90 ± 3.90 kg/m^2^, p < .05) and the role emotional domain score (yoga: 74.71 ± 35.57; control: 87.60 ± 27.56; p < 0.01, Table 1). Role emotional domain score was identified as suitable covariate to control for baseline differences on post-intervention HRQoL measures. However, analyses did not control for BMI because it was not a statistically useful covariate, as it had very weak correlations with all outcome variables. In addition, DBP and mental health component score were adjusted in MetS subgroup analyses, as they had significant correlations with various outcome measures (data not shown).

**Table 1:** Demographic Characteristics of Subjects in the Yoga Group and the Control Group (N = 173).

	Yoga (*n* = 87)	Control (*n* = 86)		
Characteristics	*M* ± *SD or n (%)*		*t or χ* ^*2*^	*P*
Age (years)	52.44 ± 7.15	51.52 ± 7.78	0.81	.422
Gender			0.33	.568
Male	34 (39.1)	30 (34.9)		
Female	53 (60.9)	56 (65.1)		
Marital status (%)			0.58	.446
Single	13 (14.9)	14 (16.3)		
Married	70 (80.5)	71 (82.6)		
Widow	4 (4.6)	1 (1.2)		
Education level (%)^a^			2.59	.108
No education	0 (0)	1 (1.2)		
Primary	5 (5.7)	5 (5.8)		
Sceondary	45 (51.7)	54 (62.8)		
Tertiary	37 (42.5)	26 (30.2)		
Occupation (%)			0.25	.615
Full-time	48 (55.2)	45 (52.3)		
Part-time	5 (5.7)	5 (5.8)		
Unemployed	0 (0)	2 (2.3)		
Housewife	18 (20.7)	12 (14.0)		
Retired	16 (18.4)	22 (25.6)		
Medication use				
*Glucose*			2.37	.124
Yes	15 (17.2)	8 (9.3)		
No	72 (82.8)	78 (90.7)		
*Blood pressure*			2.98	.084
Yes	20 (23.0)	30 (34.9)		
No	67 (77.0)	56 (65.1)		.805
*Cholesterol*			0.06	
Yes	12 (13.8)	13 (15.1)		
No	75 (86.2)	73 (84.9)		.317
*Triglycerides*			1.00	
Yes	3 (3.4)	1 (1.2)		
No	84 (96.6)	85 (98.8)		
Smoking (%)			0.06	.813
Current	1 (1.1)	2 (2.3)		
Quitted	6 (6.9)	5 (5.8)		
Never	80 (92.0)	79 (91.9)		
Alcohol intake (%)^a^			3.09	.079
Quitted	1 (1.1)	1 (1.2)		
Never	30 (34.5)	43 (50.0)		
Sometimes	53 (60.9)	39 (45.3)		
Always	3 (3.4)	3 (3.5)		
Physical activity (MET-minutes/week)	1796.33 ± 1817.63	1701.91 ± 1430.95	0.35	.724
Vigorous physical activities (days/week)	1.05 ± 1.45	0.71 ± 1.34	1.59	.115
Moderate physical activities (days/week)	1.46 ± 1.92	1.52 ± 1.79	-0.23	.822
Walking (days/week)	5.61 ± 1.96	5.81 ± 1.73	-0.73	.467
Body weight (kg)	64.68 ± 11.60	67.99 ± 11.74	-1.87	.063
Body mass index (kg/m^2^)	24.44 ± 3.84	25.90 ± 3.90	-2.48	.014*
Metabolic risk factors				
WC, cm	87.43 ± 10.37	90.20 ± 9.93	-1.80	.074
SBP, mmHg	128.59 ± 16.12	132.17 ± 20.64	-1.27	.205
DBP, mmHg	77.30 ± 10.54	80.59 ± 11.74	-1.94	.054
FBG, mmol/L	5.63 ± 0.90	5.67 ± 1.23	-0.24	.808
TG, mmol/L	1.46 ± 0.96	1.50 ± 1.12	-0.28	.778
HDL-C, mmol/L	1.49 ± 0.41	1.45 ± 0.40	0.65	.520
MetS z score	-1.07 ± 3.16	-0.27 ± 3.45	-1.61	.110
Health-related quality of life				
Physical functioning (PF)	88.74 ± 10.57	85.76 ± 12.31	1.71	.089
Role physical (RP)	82.76 ± 28.86	85.76 ± 26.16	-0.72	.475
Bodily pain (BP)	65.97 ± 19.91	67.42 ± 22.32	-0.45	.652
General health perceptions (GH)	60.69 ± 11.91	60.99 ± 12.00	-0.16	.870
Physical component score (PCS)	298.15 ± 53.50	299.92 ± 53.90	-0.22	.829
Vitality (VT)	66.78 ± 15.06	65.58 ± 16.88	0.49	.622
Social functioning (SF)	84.48 ± 18.08	87.94 ± 16.21	-1.32	.188
Role emotional (RE)	74.71 ± 35.57	87.60 ± 27.56	-2.67	.008**
General mental health (MH)	75.26 ± 13.85	77.91 ± 14.20	-1.24	.217
Mental component score (MCS)	301.24 ± 68.09	319.02 ± 61.23	-1.81	.073

The total percentage does not sum to exactly 100% due to rounding.

WC waist circumference; SBP systolic blood pressure; DBP diastolic blood pressure; FBG fasting blood glucose; TG triglyceride; HDL-C high-density lipoprotein cholesterol; MetS Metabolic syndrome

^a^The total percentage does not sum to exactly 100% due to round-off error.

*p < 0.05,

**p < 0.01.

### Changes in metabolic risk factors

Analysis involving the entire cohort revealed that the yoga group achieved greater decline in waist circumference (-2.64 ± 2.56 cm), compared with the control group (-0.20 ± 2.19 cm) (p < 0.001, partial η^2^ = 0.209). The yoga group also showed greater reduction in fasting glucose (-0.14 ± 0.40 mmol/L) than the control group (0.05 ± 0.36 mmol/L) (p < 0.01, partial η^2^ = 0.056). However, the effect for triglycerides was modest (p 0< 0.05, partial η^2^ = 0.026). No significant differences between groups were observed in the mean change of SBP/DBP or HDL-C (all p > 0.05). With regards to MetS z score, the yoga group demonstrated significantly greater reductions (-0.79 ± 1.26) than the control group (-0.19 ± 0.99) (p 0< .01, partial η^2^ = 0.067, Table 2). Waist circumference (p = 0.022) and HDL-C (p = 0.020) were the only measures for which the treatment arm-by-MetS subgroup interaction terms were statistically significant (data not shown).

**Table 2:** Comparison of changes of outcome variables between the yoga group and the control group (N = 173).

	Yoga (*n* = 87)			Control (*n* = 86)			ANOVA		ANCOVA	
	Pre	Post	Post—Pre	Pre	Post	Post—Pre		Effect size	Adjusted	Effect size
Outcome variables	*M* ± *SD*	*M* ± *SD*	*M* ± *SD*	*M* ± *SD*	*M* ± *SD*	*M* ± *SD*	*p*	partial η^2^	*P*	partial η^2^
*Metabolic risk factors*										
WC, cm	87.43 ± 10.37	84.79 ± 10.02	-2.64 ± 2.56	90.20 ± 9.93	90.00 ± 10.05	-0.20 ± 2.19	.000***	0.209	/	/
SBP, mmHg	128.59 ± 16.12	124.30 ± 15.10	-4.29 ± 10.05	132.17 ± 20.64	130.13 ± 19.83	-2.05 ± 12.60	.197	0.010	/	/
DBP, mmHg	77.30 ± 10.54	76.47 ± 9.03	-0.83 ± 6.66	80.59 ± 11.74	79.47 ± 11.97	-1.13 ± 7.76	.785	0.000	/	/
FBG, mmol/L	5.63 ± 0.90	5.49 ± 0.89	-0.14 ± 0.40	5.67 ± 1.23	5.71 ± 1.18	0.05 ± 0.36	.002**	0.056	/	/
TG, mmol/L ^†^	1.46 ± 0.96	1.28 ± 0.64	-0.18 ± 0.70	1.50 ± 1.12	1.51 ± 1.11	0.01 ± 0.44	.033*	0.026	/	/
HDL-C, mmol/L	1.49 ± 0.41	1.52 ± 0.39	0.03 ± 0.18	1.45 ± 0.40	1.50 ± 0.40	0.05 ± 0.17	.594	0.002	/	/
MetS z score	-1.07 ± 3.16	-1.86 ± 2.87	-0.79 ± 1.26	-0.27 ± 3.45	-0.45 ± 3.44	-0.19 ± 0.99	.001**	0.067	/	/
*Health-related quality of life*										
Physical functioning (PF)	88.74 ± 10.57	90.06 ± 10.55	1.32 ± 7.83	85.76 ± 12.31	85.47 ± 11.82	-0.29 ± 11.33	.277	0.007	/	/
Role physical (RP)	82.76 ± 28.86	84.20 ± 25.89	1.44 ± 32.20	85.76 ± 26.16	79.36 ± 33.47	-6.40 ± 26.62	.083	0.017	/	/
Bodily pain (BP)	65.97 ± 19.91	68.01 ± 19.56	2.05 ± 17.81	67.42 ± 22.32	64.24 ± 20.79	-3.17 ± 20.23	.073	0.019	/	/
General health perceptions (GH)	60.69 ± 11.91	62.18 ± 12.17	1.49 ± 9.89	60.99 ± 12.00	58.55 ± 12.17	-2.44 ± 9.32	.008**	0.041	/	/
Physical component score (PCS)	298.15 ± 53.50	304.45 ± 49.74	6.30 ± 46.40	299.92 ± 53.90	287.62 ± 57.87	-12.30 ± 41.47	.006**	0.043	/	/
Vitality (VT) ^‡^	66.78 ± 15.06	70.40 ± 14.25	3.62 ± 13.50	65.58 ± 16.88	66.86 ± 15.57	1.28 ± 12.03	.230	0.008	.545	0.002
Social functioning (SF) ^‡^	84.48 ± 18.08	88.94 ± 13.40	4.45 ± 17.88	87.94 ± 16.21	82.99 ± 18.68	-4.94 ± 15.61	.000***	0.073	.003**	0.052
Role emotional (RE) ^‡^	74.71 ± 35.57	81.61 ± 31.24	6.90 ± 35.99	87.60 ± 27.56	86.43 ± 28.18	-1.16 ± 29.13	.107	0.015	.919	0.000
General mental health (MH) ^‡^	75.26 ± 13.85	78.07 ± 12.63	2.80 ± 11.78	77.91 ± 14.20	76.88 ± 13.01	-1.02 ± 12.24	.038*	0.025	.120	0.014
Mental component score (MCS) ^‡^	301.24 ± 68.09	319.02 ± 56.62	17.78 ± 61.80	319.02 ± 61.23	313.17 ± 61.47	-5.85 ± 50.26	.006**	0.043	.109	0.015

Pre baseline; Post post-intervention; Post-Pre change from baseline to post-intervention; η^2^ eta-squared; WC waist circumference; SBP systolic blood pressure; DBP diastolic blood pressure; FBG fasting blood glucose; TG triglyceride; HDL-C high-density lipoprotein cholesterol; MetS Metabolic syndrome.

The effect size in ANOVA as represented by value of partial eta squared (η^2^): very small (< 0.01), small (0.01–0.05), medium (0.06–0.13), and large (≥ 0.14).

^†^ Log 10 reverse-transformation was applied to reduce the skewness of negatively skewed outcome variable.

^‡^ Adjusted for role emotional domain at baseline.

*p < 0.05;

**p < 0.01;

***p < 0.001

Subsequent subgroup analyses revealed that both the MetS and non-MetS yoga subgroups showed greater decline in waist circumference (MetS: adjusted p < 0.001, partial η^2^ = 0.287; non-MetS: p < 0.01; partial η^2^ = 0.111) and MetS z score (MetS: p < 0.01, partial η^2^ = 0.081; non-MetS: p < 0.05, partial η^2^ = 0.055), compared with the control groups. However, there were no statistically significant differences in the mean change of waist circumference and MetS z score between the MetS subgroups (within yoga: MetS vs. non-MetS, p = 0.087 for waist circumference, p = 0.158 for MetS z score, data not shown). The non-MetS yoga subgroup also showed greater decline in SBP (p < 0.05, partial η^2^ = 0.049) and fasting glucose (p < 0.01, partial η^2^ = 0.098), compared with the control group. No significant differences were found for other metabolic risk factors between groups (all p > 0.05, Tables 3 and 4).

**Table 3:** Comparison of changes of outcome variables among MetS participants in the yoga group versus the control group (N = 87).

	Yoga (*n* = 44)			Control (*n* = 43)			ANOVA		ANCOVA	
	Pre	Post	Post—Pre	Pre	Post	Post—Pre		Effect size	Adjusted	Effect size
Outcome variables	*M* ± *SD*	*M* ± *SD*	*M* ± *SD*	*M* ± *SD*	*M* ± *SD*	*M* ± *SD*	*p*	partial η^2^	*p*	partial η^2^
*Metabolic risk factors*										
WC, cm ^†^	93.11 ± 9.63	90.01 ± 9.63	-3.10 ± 2.78	94.70 ± 7.55	94.85 ± 7.21	0.15 ± 1.99	.000***	0.316	.000***	0.287
SBP, mmHg ^†^	134.09 ± 14.23	131.16 ± 12.28	-2.93 ± 10.48	140.19 ± 20.21	136.95 ± 19.87	-3.23 ± 13.36	.907	0.000	.690	0.002
DBP, mmHg ^†^	80.45 ± 8.63	80.27 ± 7.69	-0.18 ± 6.11	85.47 ± 11.71	84.14 ± 12.22	-1.33 ± 8.67	.478	0.006	.883	0.000
FBG, mmol/L	5.94 ± 0.74	5.80 ± 0.85	-0.14 ± 0.43	6.17 ± 1.40	6.18 ± 1.31	0.01 ± 0.41	.101	0.031	/	/
TG, mmol/L ^‡^	1.87 ± 1.13	1.60 ± 0.66	-0.28 ± 0.93	1.96 ± 1.34	1.94 ± 1.35	-0.02 ± 0.54	.117	0.029	/	/
HDL-C, mmol/L	1.25 ± 0.25	1.33 ± 0.26	0.08 ± 0.18	1.28 ± 0.33	1.31 ± 0.32	0.03 ± 0.13	.161	0.023	/	/
MetS z score	1.12 ± 1.94	0.14 ± 1.76	-1.98 ± 1.47	1.97 ± 2.89	1.75 ± 2.99	-0.22 ± 1.07	.008**	0.081	/	/
*Health-related quality of life*										
Physical functioning (PF)	87.95 ± 11.33	87.95 ± 11.73	0.00 ± 7.62	85.00 ± 11.02	83.95 ± 12.70	-1.05 ± 10.03	.585	0.004	/	/
Role physical (RP)	83.52 ± 30.95	85.80 ± 25.52	2.27 ± 34.45	84.30 ± 28.36	78.49 ± 33.89	-5.81 ± 26.07	.221	0.018	/	/
Bodily pain (BP)	65.45 ± 22.65	63.14 ± 21.12	-2.32 ± 17.59	66.33 ± 21.67	62.58 ± 21.46	-3.74 ± 14.86	.684	0.002	/	/
General health perceptions (GH) ^†^	58.18 ± 12.11	60.45 ± 13.07	2.27 ± 10.54	59.30 ± 10.83	57.44 ± 13.20	-1.86 ± 9.00	.053	0.043	.129	0.027
Physical component score (PCS)	295.11 ± 59.28	297.34 ± 52.43	2.23 ± 50.05	294.93 ± 54.59	282.47 ± 61.64	-12.47 ± 41.38	.140	0.025	/	/
Vitality (VT) ^†^	65.68 ± 14.89	69.77 ± 14.94	4.09 ± 11.43	65.12 ± 15.83	65.81 ± 16.87	0.70 ± 11.26	.167	0.022	.391	0.009
Social functioning (SF) ^†^	82.67 ± 20.92	88.64 ± 12.30	5.97 ± 20.44	84.59 ± 17.43	79.36 ± 18.68	-5.23 ± 13.70	.004**	0.095	.015*	0.069
Role emotional (RE) ^†^	74.24 ± 37.95	79.55 ± 32.32	5.30 ± 38.00	84.50 ± 29.41	86.05 ± 27.44	1.55 ± 28.13	.603	0.003	.823	0.001
General mental health (MH) ^†^	74.73 ± 16.32	77.55 ± 14.05	2.82 ± 11.43	78.05 ± 13.53	75.81 ± 14.29	-2.23 ± 13.10	.059	0.041	.176	0.022
Mental component score (MCS) ^†^	297.32 ± 75.89	315.50 ± 58.66	18.18 ± 64.23	312.25 ± 63.84	307.03 ± 64.25	-5.22 ± 54.04	.070	0.038	.227	0.014

Pre baseline; Post post-intervention; Post-Pre change from baseline to post-intervention; η^2^ eta-squared; WC waist circumference; SBP systolic blood pressure; DBP diastolic blood pressure; FBG fasting blood glucose; TG triglyceride; HDL-C high-density lipoprotein cholesterol; MetS Metabolic syndrome.

The effect size in ANOVA as represented by value of partial eta squared (η^2^): very small (< 0.01), small (0.01–0.05), medium (0.06–0.13), and large (≥ 0.14).

^†^ Adjusted for DBP at baseline.

^‡^ Log 10 reverse-transformation was applied to reduce the skewness of negatively skewed outcome variable.

* *p* < 0.05,

**p < 0.01,

***p < 0.001

**Table 4:** Comparison of changes of outcome variables among non-MetS participants in the yoga group versus the control group (N = 86).

	Yoga (*n* = 43)			Control (*n* = 43)			ANOVA		ANCOVA	
	Pre	Post	Post—Pre	Pre	Post	Post—Pre		Effect Size^a^	Adjusted	Effect Size^a^
Outcome variables	*M* ± *SD*	*M* ± *SD*	*M* ± *SD*	*M* ± *SD*	*M* ± *SD*	*M* ± *SD*	*p*	partial η^2^	*p*	partial η^2^
*Metabolic risk factors*										
WC, cm	81.61 ± 7.53	79.45 ± 7.27	-2.16 ± 2.25	85.71 ± 10.06	85.15 ± 10.21	-0.56 ± 2.33	.002**	0.111	/	/
SBP, mmHg	122.95 ± 16.14	117.28 ± 14.59	-5.67 ± 9.52	124.16 ± 17.94	123.30 ± 17.48	-0.86 ± 11.82	.041*	0.049	/	/
DBP, mmHg	74.07 ± 11.41	72.58 ± 8.71	-1.49 ± 7.18	75.72 ± 9.65	74.79 ± 9.78	-0.93 ± 6.83	.713	0.002	/	/
FBG, mmol/L	5.31 ± 0.95	5.18 ± 0.84	-0.13 ± 0.36	5.17 ± 0.78	5.25 ± 0.81	0.08 ± 0.31	.003**	0.098	/	/
TG, mmol/L ^†^	1.03 ± 0.47	0.95 ± 0.43	-0.08 ± 0.32	1.05 ± 0.57	1.09 ± 0.54	0.04 ± 0.31	.077	0.037	/	/
HDL-C, mmol/L	1.73 ± 0.40	1.71 ± 0.41	-0.02 ± 0.18	1.62 ± 0.39	1.68 ± 0.38	0.06 ± 0.20	.066	0.040	/	/
MetS z score	-3.32 ± 2.53	-3.91 ± 2.28	-0.60 ± 0.96	-2.50 ± 2.35	-2.65 ± 2.28	-0.15 ± 0.91	.030*	0.055	/	/
*Health-related quality of life*										
Physical functioning (PF) ^‡^ ^||^	89.53 ± 9.81	92.21 ± 8.82	2.67 ± 7.89	86.51 ± 13.56	86.98 ± 10.81	0.47 ± 12.57	.332	0.011	.513	0.005
Role physical (RP)	81.98 ± 26.91	82.56 ± 26.47	0.58 ± 30.12	87.21 ± 24.01	80.23 ± 33.43	-6.98 ± 27.45	.227	0.017	/	/
Bodily pain (BP)	66.49 ± 16.92	73.00 ± 16.62	6.51 ± 17.11	68.51 ± 23.15	65.91 ± 20.20	-2.60 ± 24.63	.049*	0.045	/	/
General health perceptions (GH)	63.26 ± 11.28	63.95 ± 11.05	0.70 ± 9.23	62.67 ± 12.97	59.65 ± 11.09	-3.02 ± 9.71	.072	0.038	/	/
Physical component score (PCS) ^§^	301.26 ± 47.36	311.72 ± 46.31	10.47 ± 42.53	304.91 ± 53.38	292.77 ± 54.07	-12.14 ± 42.05	.015*	0.068	.048*	0.046
Vitality (VT) ^||^	67.91 ± 15.32	71.05 ± 13.65	3.14 ± 15.47	66.05 ± 18.05	67.91 ± 14.28	1.86 ± 12.86	.678	0.002	.794	0.001
Social functioning (SF) ^¶^	86.34 ± 14.64	89.24 ± 14.58	2.91 ± 14.90	91.28 ± 14.31	86.63 ± 18.17	-4.65 ± 17.47	.034*	0.053	.072	0.038
Role emotional (RE) ^||^	75.19 ± 33.41	83.72 ± 30.32	8.53 ± 34.19	90.70 ± 25.54	86.82 ± 29.22	-3.88 ± 30.18	.078	0.036	.688	0.002
General mental health (MH) ^||^	75.81 ± 10.94	78.60 ± 11.14	2.79 ± 12.27	77.77 ± 15.00	77.95 ± 11.66	0.19 ± 11.35	.310	0.012	.350	0.011
Mental component score (MCS) ^||^	305.25 ± 59.69	322.62± 54.92	17.36 ± 59.96	325.79 ± 58.47	319.31 ± 58.68	-6.48 ± 46.82	.043*	0.048	.206	0.019

*Pre* baseline; *Post* post-intervention; *Post-Pre* change from baseline to post-intervention; *η* ^*2*^ eta-squared; *WC* waist circumference; *SBP* systolic blood pressure; *DBP* diastolic blood pressure; *FBG* fasting blood glucose; *TG* triglyceride; *HDL-C* high-density lipoprotein cholesterol; *MetS* Metabolic syndrome.

The effect size in ANOVA as represented by value of partial eta squared (η^2^): very small (< 0.01), small (0.01–0.05), medium (0.06–0.13), and large (≥ 0.14).

^†^ Log 10 reverse-transformation was applied to reduce the skewness of negatively skewed outcome variable.

^‡^ Square root transformation was adopted to reduce the skewness of positive-skewed outcome variable.

^§^ Adjusted for role emotional domain score at baseline.

^||^ Adjusted for role emotional domain score at baseline and metal component score at baseline.

^¶^ Adjusted for role metal component score at baseline.

* *p* < 0.05,

**p < 0.01

### Changes in health-related quality of life

There were significant differences in both general health perceptions domain score (yoga: 1.49 ± 9.89, control: -2.44 ± 9.32, p < 0.01, partial η^2^ = 0.041) and physical component score (yoga: 6.30 ± 46.40, control: -12.30 ± 41.47, p < 0.01, partial η^2^ = 0.043) between groups. The yoga group also demonstrated greater improvements in social functioning domain score (4.45 ± 17.88) than the control group (-4.94 ± 15.61) (p < 0.001, partial η^2^ = 0.073). After adjusting for role emotional domain score at baseline, yoga intervention exerted a small effect on the social functioning domain score (adjusted p < 0.01, partial η^2^ = 0.052). None of the other HRQoL domains measure was significantly differed between groups (all p > 0.05, Table 2).

The MetS yoga subgroup showed greater improvements in the social functioning domain score (yoga: 5.97 ± 20.44, control group: -5.23 ± 13.70, p < 0.01, partial η^2^ = 0.095). Results remained significant after adjusting for DBP at baseline (adjusted p < 0.05, partial η^2^ = 0.069). No differences were found for general health perceptions, physical component score, or other domains score between groups (all p > 0.05, Table 3). However, the non-MetS yoga subgroup did not show any improvement in social functioning domain score (p > 0.05) but demonstrated significant differences in the mean change of bodily pain domain score and physical component score (both p < 0.05), compared with the control groups (Table 4).

### Adherence, acceptability, and feasibility

The overall adherence rate was 89% (yoga group: 91.8%, control group: 87.2%) and the mean attendance rate of 12 sessions was 93.6%. According to the log records for practice at home, 99% of the yoga participants practiced yoga at home for an average of about 165 minutes per week (23 minutes per day). No unanticipated adverse events were reported among participants.

## Discussion

The present study demonstrated that 12 weeks of Hatha yoga training resulted in significant beneficial effects on metabolic risk factors (including waist circumference, fasting glucose, and triglycerides), MetS z score, and HRQoL (including general health perceptions, social functioning, and physical health component score) compared with the control group. Yoga training reduced waist circumference and MetS z score, regardless of whether participants had MetS or not.

Although the yoga training in our study was relatively less intensive (12 weekly 6-minute session), our finding of a significant reduction in waist circumference is consistent with results reported by Littman *et al*.[28] and Lee *et al*.[29] who showed that waist circumference was reduced significantly after moderate-to-intensive yoga training (3–5 times per week for 16 weeks-6 months) in obese women. While the mechanisms responsible for this change cannot be directly determined in this study, it is possible that some yoga poses may have achieved optimal intensity for fat metabolism and weight loss.[11]

The significant reduction in fasting glucose in the total cohort and non-MetS subgroup in this study also confirm the findings of several previous yoga intervention studies with regards to improving fasting glucose in obese or diabetic individuals,[29, 30] and a previous review of 25 yoga studies,[15] which concluded that practicing yoga may have a positive effect on fasting glucose. The underlying mechanisms between yoga and its influence of fasting glucose have not been fully delineated. It is possible that by reducing the activation activity of the sympathoadrenal system (SAS) and the hypothalamic pituitary adrenal (HPA) axis, yoga may reduce perceived stress, enhance psychological well-being, and foster multiple positive downstream effects on neuroendorince status and metabolic function,[31, 32] and thus reduce blood glucose levels. Alternatively, yoga may decrease oxidative stress and improve antioxidant status, and thereby exerting protective effects on type 2 diabetes.[30] However, the apparent beneficial effects of yoga for fasting glucose was not found in MetS participants in the present study; further study is needed to clarify the glucose-lowering effect of yoga training.

The modest effect on triglycerides in the present study was limited to the total cohort and was not observed in the subgroup analyses, probably as a consequence of the reduced sample size. No significant differences in HDL-C were found between the yoga and control group, in contrast to Lee *et al*. study,[29] in which a 16-week yoga program appeared to offer benefits in serum lipids (including triglycerides and HDL-C) in obese postmenopausal women. The absence of a significant difference in HDL-C in the present study may in part due to the moderately high baseline levels of HDL-C (1.5 mmol/L), which may reduce the statistical power of this analysis in demonstrating the intervention effect on this outcome. It is also possible that the level of yoga training of the present subjects was not sufficient to induce increase in HDL-C. However, a recent yoga study employing a vigorous style of yoga (3 times weekly Bikram yoga) also failed to show an increase in HDL-C.[33] We speculate that an increase in HDL-C may require a longer duration of training to be elicited, particularly for the individuals with high baseline HDL-C levels.

Our findings also demonstrated a small favorable effect of the yoga program on SBP in the non-MetS subgroup but not in the total cohort or MetS subgroup. The reason for this observation is unclear. It is possible that anti-hypertensive medication use (with the prevalence rate of 41%) in MetS yoga participants attenuated the potential beneficial effects of yoga training on their blood pressure levels. The underlying mechanism for the effect of yoga on SBP in the non-MetS subgroup is also uncertain, but may be similar to the effects of deep breathing exercises, which exert their blood pressure lowering effect by increasing the sensitivity of baroreflex (a homeostatic process that helps to maintain blood pressure).[34] Device-guided slow breathing has been suggested as a nonpharmacological approach to the treatment of hypertension.[35] Mindfulness-based stress reduction, which incorporate slow and deep breathing techniques, have also been found to contribute to lowering of blood pressure.[36]

Although yoga may improve individual risk factors of MetS, there is little research on the effect of yoga on MetS as a whole. To our knowledge, only a few studies specifically targeted individuals with MetS, with significant improvements in metabolic parameters.[37, 38] Taking into account the concept of the MetS z score, the present study can infer that yoga training was effective in improving the MetS z score, in both MetS and non-MetS yoga participants. The decrease in waist circumference and fasting glucose levels coupled with the modest reduction of triglycerides observed in the yoga group after the intervention could have contributed to the decrease in MetS z score. Thus, our findings extended the existing data by establishing that yoga training positively impact metabolic risk score in Chinese adults.

Apart from metabolic risk, it would be of interest to explore the effects of yoga on HRQoL, since MetS and its components have been associated with worse HRQoL.[39] The present study found concurrent improvement in certain aspects of HRQoL per the SF-36 measure, including bodily pain, general health perceptions, physical component score, and social functioning. Prior trials showed improved quality of life for people with hypertension or heart failure who completed a supervised yoga program.[40, 41] We add to these prior studies by showing improved quality of life for people (even with MetS) and who participated in a 12-weeks yoga intervention.

The improvements in general health perceptions and physical component score could be considered from different perspectives. First, yoga participants may have experienced the genuine benefits of yoga practice (e.g., improved flexibility and fitness). Second, the placebo effect may have a significant impact, in particular when the intervention is non-blinded.[42] A recent survey has demonstrated that participants believe yoga could improve their health, regardless of their health status.[43] These findings are noteworthy because self-perceived physical health may powerfully predict an individual’s capacity to manage the disease, which in turn may result in improved metabolic profile. A recent study also showed that self-perceived physical health is a significant independent predictor of cardiovascular disease incidence and mortality among postmenopausal women.[44] However, our findings showed that bodily pain was improved in the non-MetS subgroup only. Differences in the baseline characteristics may be one of the possible reasons for this discrepant result between the two subgroups.

The findings of the improved social functioning is not unexpected given the small-group setting of yoga intervention may have provided socialization opportunities for the participants. Considering that socialization is an important factor correlated with further health outcomes,[45] the improvement in social functioning is important, particular for those who are already at risk. However, the mental sub-scores failed to reach statistical significance in the present study. It is possible that the importance of physical fitness is the most emphasized in Hatha yoga from all yoga traditions, thus limited its effects on psychological outcomes. Furthermore, as participants had relatively low levels of perceived stress or mood symptoms at the baseline (data not shown), this limited interpretation of the impact of yoga intervention in this group of participants.

Qualitative analyses of feedback from participants suggest that this form of treatment was well received, with most of them believed yoga is helpful for enhancing their health. Although most of the participants reported some difficulties with some of the yoga poses at the beginning of the intervention, this did not influence class attendance. Furthermore, compliance was good with most of the participants reported self-participation of yoga at home. In view of the high prevalence of physical inactivity, our results suggest that Hatha yoga may be an alternative training modality for health enhancements.

The limitations of this study include the lack of randomization, which may have led to selection bias and decreased comparability between groups with various confounding factors. Other limitations include non-blinded assessment of outcomes, the self-report measures of HRQoL, and the possible lack of measures of confounding factors such as diet and sleep quality. In addition, the participants were a highly motivated group that was willing to volunteer for a research study, and the yoga class was adapted for beginners, therefore findings may not be directly generalizable to a typical community yoga class. Finally, because the yoga classes were offered to participants once per week, the frequency of sessions may be different from that of other intervention studies, which makes comparison between studies difficult. However, our study has several notable strengths including the use of an expert yoga practitioner to design a program specifically for middle-aged to older adults, a sex-balanced stratified sample according to the presence of MetS, and the inclusion of multiple outcome variables.

In conclusion, this study showed that a 12-week Hatha yoga program for Chinese adults produced beneficial changes in waist circumference, fasting glucose, triglycerides, MetS z score, and HRQoL. The reductions in waist circumference and MetS z score did not differ between MetS subgroups. Given the low-to-moderate intensity feature of yoga, the high adherence rate, and the encouraging results of this study, yoga may be a promising alternative form of exercise for Chinese individuals with or without MetS. However, yoga was not beneficial in improving HDL-C in this short-term study. Further investigation with longer follow-up (e.g., 6 months) should be considered, which would offer insights as to the long-term benefits of yoga.

## Supporting Information

S1 Fig Recruitment of subjects. (DOCX)

S1 File Research protocol. (DOC)

S2 File Trend statement checklist. (PDF)
