# Supplementary material for: Effects of a 12-Week Hatha Yoga Intervention on Metabolic Risk and Quality of Life in Hong Kong Chinese Adults with and without Metabolic Syndrome
Source: PLoS One. 2015 Jun 25;10(6):e0130731. doi: 10.1371/journal.pone.0130731 (PMC4482438; doi:10.1371/journal.pone.0130731)
Supplement: S1 Fig — (DOCX) [file pone.0130731.s001.docx]

Supporting Information Figure S1

**Figure 1 Recruitment of subjects**
