# Supplementary material for: Effects of a 12-Week Hatha Yoga Intervention on Metabolic Risk and Quality of Life in Hong Kong Chinese Adults with and without Metabolic Syndrome
Source: PLoS One. 2015 Jun 25;10(6):e0130731. doi: 10.1371/journal.pone.0130731 (PMC4482438; doi:10.1371/journal.pone.0130731)
Supplement: S1 File — (DOC) [file pone.0130731.s002.doc]

S1 File

**Scientific and historical background to the Study**

Prevalence of Metabolic Syndrome (MetSyn)

The metabolic syndrome (MetSyn) represents a constellation of risk factors that include abdominal obesity, hypertension, dyslipidemia, and insulin resistance, multiple risk factors for cardiovascular diseases (CVD) (Reaven, 1988). This disorder is highly influenced by both genetic and lifestyle. Several definitions of the metabolic syndrome have been proposed including those of the World Health Organization (WHO), the European Group for Study of Insulin Resistance (EGIR), the National Cholesterol Education Program (NCEP) Adult Treatment Panel III (ATP III), the American Association of Clinical Endocrinologist (AACE), and the International Diabetes Federation (IDF) (Grundy et al., 2005). The inclusion criteria from ATP III have been widely used due to its clinical simplicity, and satisfy both clinical and research needs and allow better comparisons between different study populations (Morley & Sinclair, 2009; Sullivan, 2006; Tan, Chew, Ma, Tai, & Wai, 2004; Tong et al, 2007). According to revised ATP III clinical diagnosis criteria for Asian populations, except for Japan in waist circumference an individual was deemed to have MetSyn when three or more of the following five criteria were satisfied: 1) waist circumference (WC) ≥ 90 cm in men and ≥ 80 cm in women; 2) triglyceride level ≥ 150 mg/dL or on drug treatment for elevated triglyceride; 3) high-density lipoprotein cholesterol (HDL-C) < 40 mg/dL in men, < 50 mg/dL in women or on drug treatment for reduced HDL-C; 4) systolic BP ≥ 130 mmHg or diastolic BP ≥ 85 mmHg or on antihypertensive drug treatment in a patient with a history of hypertension; and 5) fasting glucose level > 110 mg/dL or on drug treatment for elevated glucose (Grundy et al., 2005). Using the aforementioned revised ATP III criteria, the prevalence of MetSyn was 16.7% of the Hong Kong population aged 25-74 years, and the prevalence of having at least 2, 3, 4 or 5 components was 34.5%, 16.7%, 6.4% and 1.4%, respectively (Thomas et al., 2005). With the high prevalence of MetSyn in adult Hong Kong population, actions should be taken in order to control the alarming situation.

Contributing Factors of MetSyn

Theories have been suggested to explain the contributing factors of MetSyn. Stress, may lead to anxiety and depression in general, is one of the contributing factors for MetSyn. It has been known for a long time that the hypothalamic-pituitary-adrenal (HPA) axis can respond sensitively to external stimulation. A variety of agents and treatments (called stressors) are able to override the feedback systems, leading to enhanced frequency and amplitude of cortisol pulse (Björntorp & Rosmond, 2000; Kirschbaum & Hellhammer, 1989). Stress activates the sympathoadrenal system and results in chronically elevated cortisol levels and sympathetic tone (Fowler, Moussouttas, & Mancini, 2005). This association thought to be mediated by dysregulation of the HPA axis and sympathoadrenal system. There is growing evidence that these negative affective states, in turn, increase risk for visceral obesity, hypertension, insulin resistance, and other features of insulin resistance syndrome (IRS), as well as for CVD and diabetes. Moreover, negative emotional states and responses, once entrenched, may themselves operates as profound sources of psychogenic stress, contributing to chronic HPA axis activation and aversive neural programming. A vicious cycle is thus initiated resulting in progressive emotional, cognitive, structural, and physiologic impairment and culminates in the development and progression of CVD (Björntorp & Rosmond, 1999; Björntorp & Rosmond, 2000; Chrousos, 2000; Innes, Vincent, & Taylor, 2007; Kyrou, Chrousos, & Tsigos, 2006; Rosmond, 2005; Tsiogos & Chrousos, 2002).

Sedentary lifestyle or physical inactivity is another contributing factor for MetSyn, and low cardiovascular fitness level has been associated with development of MetSyn independent of obesity and other important confounding factors. Physical inactivity is a major underlying risk factor for chronic heart disease, and enhances the lipid and nonlipid risk factors of the MetSyn. It may exacerbate the risk by reducing cardiovascular fitness and coronary blood flow. Physical inactivity is associated with insulin resistance and subsequent diabetes (Fowler et al., 2005; Mittal, 2008).

Potential Prevention and Treatment of MetSyn

MetSyn is therefore greatly influenced by lifestyle. Since there is no predefined standard of treatment for MetSyn, appropriate lifestyle modifications are essential in preventing and treating MetSyn. Stress management and regular physical activity are key approaches to both prevent and treat MetSyn. Alleviating stress and improving mental health can help reduction of the aforementioned dysregulation of the HPA axis and sympathoadrenal system (Chrousos, 2000; Fowler et al., 2005; Innes, Vincent, & Taylor, 2007). Physical activity has been shown to decrease the risk and lengthen the development of MetSyn by improving MetSyn risk factors (Franks, Ekelund, Brage, Wong, & Wareham, 2004; Hwang, Bai, Chen, & Chien, 2007; Johnson et al., 2007; Rennie, McCarthy, Yazdgerdi, Marmot, & Brunner, 2003; Roberts-Grey, 2008). Yoga practice is traditionally believed to have physical and psychological benefits, and well accepted as a kind of mind-body therapy and recreational physical activity. It is therefore worth investigating the influence of yoga practice on prevention and treatment of MetSyn.

Basic Information About Yoga

Yoga originated in Indian more than 5000 years ago, its principle is to achieve the integration of body, mind, and spirit. The type of yoga generally practiced in modern, and especially Western society, is “hatha” which focuses on the path toward enlightenment and wellness through physical, mental, and spiritual means (Kappmeier & Ambrosini, 2006). Hatha yoga consists of three basic components, namely “asanas” (yogic poses), “pranayama” (breathing exercises) and “Dhyana” (meditation). The postures involve standing, bending, twisting and balancing the body, and improve flexibility and strength in controlled fashion. The controlled breathing helps mind focus and is an important component of relaxation, a modulator of autonomic nervous system function. Meditation aims to calm the mind. These three components are integrated with one another (Riley, 2004).

Benefit of Regular Yoga Practice

Although yoga has its origins in Indian culture, it has been increasingly popular as a kind of recreation or physical activity (Saper, Eisenberg, Davis, Culpepper, & Phillips, 2004), and its physical and psychological benefits have been well marketed to different populations such as the pregnant women (Beddoe, Yang, Kennedy, Weiss, & Lee, 2009), menopausal women and those with menopausal symptoms (Booth-LaForce, Thurston, & Talyor, 2007; Elasvsky & McAuley, 2007; Innes, Selfe, & Talyor, 2008; Lee, Kim, Ha, Boddy & Ernst, 2009), and the elderly (Chen et al., 2008; Kraemer & Marquez, 2009), as an alternative therapeutic intervention or mind-body therapy. Western physiological explanations of how hatha yoga might be effective as a therapy focuses on the following points: 1) the modulation of autonomic nervous system tone, which commonly means a reduction in sympathetic tone; 2) the simultaneous activation of antagonistic neuromuscular systems such as flexion and extension and intrasfusal and golgi tendon-organ feedback may provide a way to maintain range of motion and increased the relaxation response in the neuromuscular system; and 3) these kinds of meditative exercises seems to be able to stimulate the limbic system (Riley, 2004).

Yoga Practice as an Alternative and Complementary Therapy

In recent years publications showed that yoga had been applied to manage and reduce the symptoms of individuals who were suffering urologic disorders (Ripoll & Mahowald, 2002), pulmonary tuberculosis (Visweswariah & Telles, 2004), and osteoarthritis (Bukowski, Conway, Glentz, Kurland, & Galantino, 2007). Physical benefits, decreased distress and relief of perceived pain of individuals with rheumatoid arthritis (Bosch, Traustadottir, Howard, & Matt, 2009), chronic obstructive pulmonary disease (Donesky-Curenco, Nguyen, Paul, & Carrier-Kohlman, 2009), chronic back pain (Groessl, Weingart, Aschbacher, Pada, & Naxi, 2008; Tekur, Singhow, Nagendra, & Raghuram, 2008), and even cancer (DiStasio, 2008) were also found.

Mental health problems such as depression, anxiety and mood state are amongst the most common reasons for individuals to seek treatments with complementary therapies. Consequently, several surveys have been conducted which focused on this area (Pilkington, Kirkwood, Rampes, & Richardson, 2005). Moreover, published studies indicating positive results in depression, stress, anxiety, mood state, eating disorder and insomnia of yoga intervention (Beddoe et al., 2009; Bosch et al., 2009; Galentino et al., 2004; Granath, Ingvarsson, von Thiele, & Lundberg, 2006; Khalsa, 2004; Lavey et al., 2005; Michalsen et al., 2005; Oken et al., 2006; Vadiraja et al., 2009; Woolery, Myers, Sternlieb, & Zeltzer, 2004). Yoga may also help individuals to deal with the emotional aspects of chronic pain and diseases. Recent publications in yoga for patients with cancer also showed positive effects of fatigue, sleep quality, mood, stress, and quality of life (Culos-Reed, Carlson, Daroux, & Hately-Aldous, 2006; DiStasio, 2008; Vadiraja et al., 2009).

Potential Effects of Regular Yoga Practice on MetSyn

As reviewed above, yoga has been showing positive impacts on different physical and mental health problems. Yoga may have the potential impacts on prevention and management of MetSyn. There is increasing evidence that even short-term practice of yoga can reduce both psychological and physiological risk factors for CVD and may attenuate signs, reduce complications, and improve the prognosis of clinical disease. Although the mechanisms underlying the observed beneficial effects of yoga therapy on cardiovascular risk profiles remain speculative, the observed changes likely occur through at least two pathways. First, by reducing the activation and reactivity of the sympathoadrenal system and the HPA axis and promoting feelings of well-being, yoga may alleviate the effects of stress and foster multiple positive effects on neuroendocrine status, metabolic function, and related inflammatory response. As mentioned above yoga appears to have widespread beneficial effects on both psychological and physiological indices of cardiovascular risk. These effects likely occur in part via the stabilization of HPA axis and sympathoadrenal activity. The attendant reduction in catecholamine and cortisol, decline in cardiovascular reactivity, enhancement of mood and well-being, and alleviation of perceived stress may result in positive downstream effects on metabolic and hemodynamic profiles (Innes et al., 2007). Yoga practice induces potential decrease in salivary cortisol level thus pointing to a direct effect on HPA axis. To further reveal potential mechanisms of the yoga induced physiopsychological benefits, especially stress reduction, growing body of researches estimated salivary cortisol levels to act as an objective measure of HPA axis activity. Results of various studies indicated the trend of decrease (Beddoe et al., 2009; Woolery et al., 2004) or significant decrease in cortisol levels after completion of yoga interventions in different populations (Bosch et al.,2009; Michalsen et al., 2005; West et al., 2004; Vadiraja et al., 2009).

Second, it is suggested that yoga practice (deep and slow breathing and asanas which help stretch and relax muscles) might counteract the aroused autonomic nervous system (ANS) activity, and might even reverse it back to the relaxed state (Levine, 2000; Michalsen et al., 2005). By directly stimulating the vagus nerve, yoga may enhance parasympathetic output and thereby shift the ANS balance from primarily sympathetic to parasympathetic, leading to positive changes in cardiac-vagal function, in mood and energy state, and in related neuroendocrine, metabolic, and inflammatory responses. Vagal stimulation triggers a series of autonomic responses that may buffer the effects of chronic stress and help restore sympathovagal balance. For example, vagal stimulation slows heart rate, reduces blood pressure, induces gastric motility, dilates arterioles, and constricts the pupils. Stimulation of the vagus nerve also suppresses inflammation and activates the cholinergic anti-inflammatory pathway, which inhibits synthesis of TNF-α, reduces TNF-αserum concentrations, and inhibits TNF-αactivity, leading to a reduction in proinflammatory cytokines. This anti-inflammatory effect, in turn, leads to reduce HPA axis and sympathoadrenal activation and related positive, downstream changes (Innes et al., 2007). Telles and her colleagues (2004) investigated the effect of yoga on ability to reduce the heart rate voluntarily in those who were novices to yoga practice, and the result suggested that yoga training can enable practitioners to use their own way to reduce the heart rate. The study of Khattab and colleague (2007) suggested that relaxation of yoga training is associated with significant increase of cardiac vagal modulation. Recent study examined the effect of breathing exercise on patients with hypertension also suggested that improvement in both sympathetic and parasympathetic reactivity may be the mechanism that is associated in pranayama practice (Mourya, Mahajan, Singh, & Jain, 2009). The result of study about the effect of integrated yoga practice on perceived stress and measured autonomic response in healthy pregnant women also indicated that yoga reduced stress and improves adaptive autonomic response to stress to those women (Satyapriya, Nagendra, Nagarathna, & Padmalatha, 2009). Thus, it is suggested that through regular yoga practice, we might calm ourselves in a provocative situation, and even prevent an ANS (arousal) response to a provoking situation (Levine, 2000).

Increasing physical activity is also helpful in the treatment of MetSyn. Consider yoga as a kind of physical activity, it bears the above benefit in treatment of MetSyn. Regular and sustained physical activity improves all risk factors of the MetSyn. Physical activity improves the IRS, vascular and cholesterol problems. Physical activity reduces blood pressure, insulin resistance, and favourably affect cardiovascular outcome. High levels of physical activity have quite consistently protected against the development of obesity, diabetes and cardiovascular disease, conditions that are commonly associated with the MetSyn. Even a low level of leisure-time physical activity tended to decrease the likelihood of developing the MetSyn. Recreational physical activities, rather than employment-related activities, appear to lead more closely to weight change. A greater increase in physical activity has larger effects on body composition and cardiovascular fitness (Mendelson, 2008; Mittal, 2008).

In addition, yoga has many practical advantages as lifestyle and therapeutic intervention and health promotion measures. These practices are relatively simple to learn, are economical (can be home-based and not require many equipment), noninvasive therapies with few side effects and multiple collateral lifestyle benefits. Individuals can gain social support from group practice and achieve a state of calm from individual practice. Group and individual practice may also help to improve lifestyle choices and health-related quality, in part by enhancing psychological well-being, and thereby contribute significantly to health promotion. Yoga are low impact non competitive in nature, and can be low risk and safe if individuals follow proper instructions and perform properly. Moreover, individuals can feel relax and calm right after each practice, these positive feelings can encourage individuals’ adherence on yoga practice. Yoga may also provide a good alternative to conventional exercise for elderly, ill, unfit, overweight individuals and those who experience greater barriers to initiating and continuing conventional exercise programs than others (Innes et al., 2008).

**Purpose of the Study**

Yoga practice has numerous physical and mental health benefits and considerable therapeutic potential, yet quality controlled studies investigating the effects of these practices on CVD risk factors or MetSyn remain few, especially in Asian populations. Potential long-term effects of yoga remains little explored and the mechanisms underlying the reported benefits associated with yoga are still not clearly understood. Moreover, interpretation of and comparisons across many existing studies are limited by methodological problems such as small sample sizes, insufficient description of methods, selection bias, lacking of randomization, insufficient in statistical analysis and presentation*,* and the large variation in the type, duration, intensity, and delivery methods of the interventions. Thus additional high-quality randomized controlled trials are needed to investigate beneficial effects of yoga on both physical and mental health, especially in Asana population.

**Research Objectives**

The objectives of the present study will be threefold: 1) to examine the beneficial effects of yoga practice on physical and mental health; 2) to examine the effectiveness of regular yoga practice on improvement of MetSyn and each MetSyn component; 3) and to elucidate the possible underlying mechanisms of yoga practice by which improve MetSyn.

**Null Hypotheses**

The null hypotheses formulated for this study are listed below:

1. There is no significant difference in health-related physical fitness (i.e. cardiovascular endurance, muscle strength and endurance, flexibility, body composition) before and after who participated in the hatha yoga program.
2. There is no significant difference in HRQOL before and after who participated in the hatha yoga program.
3. There is no significant difference in stress level before and after who participated in the hatha yoga program.
4. There is no significant difference in depression before and after who participated in the hatha yoga program.
5. There is no significant difference in anxiety before and after who participated in the hatha yoga program.
6. There is no significant difference in self-esteem before and after who participated in the hatha yoga program.
7. There is no significant difference in cortisol level before and after who participated in the hatha yoga program.
8. There is no significant difference in each MetSyn component (i.e. WC, triglyceride level, HDL-C, BP, and fasting glucose level) before and after who participated in the hatha yoga program.
9. There is no significant difference in MetSyn z score before and after who participated in the hatha yoga program.

**Definition of MetSyn and MetSyn z score**

The revised ATP III aforementioned in the background will used to define MetSyn in this study, and the MetSyn score will be calculated by summing the standardized values for WC, triglyceride level, HDL-C, BP, and fasting glucose level. Gender-specific z scores will be used to account for variations in ATP III criteria for men and women. Each variable will be standardized by subtracting the sample mean from the individual mean and dividing by SD. Both baseline and post intervention z scores will be calculated with same transformation. This MetSyn z score, a continuous score, will be used for the increase of statistical power as the above variables are not dichotomized. The concept of a continuous score to evaluate MetSyn was also used by Johnson, et al (2007) and Wijndaele, et al (2009) elsewhere.

**Methodology**

Instruments

In this study, the instruments with established validity and reliability will be used to determine the individual components of MetSyn, HPA axis activity, health-related physical fitness, mental health, and physical activity level of the subjects.

Parameters used to indicate the individual components of MetSyn:

1. Blood pressure: Blood pressure measurements will be measured in seated position according to the American Heart Association Scientific Statement (Pickering et al., 2005). The average of the second and third systolic and diastolic pressure reading will be used in the analyses.
2. Waist circumference (WC): WC will be measured at the nearest 0.5 cm at the shortest point below the lower rib margin and the iliac crest.
3. Blood profile (fasting glucose, triglycerides, and HDL-C): The profile will obtained by taking blood samples from subjects and sent the samples to the laboratory for analysis.

Parameter used to indicate the effect on HPA axis activity:

Physiological stress: The measurement of salivary cortisol provides a non-invasive and reliable tool for investigation of HPA axis activity. Subjects will be instructed to obtain salivary samples at the same time to control the circadian rhythm (Kirschbaum & Hellhammer, 1989). Samples will be sent to the laboratory for electrochemiluminescence immunoassay. Raw cortisol values will be log transformed to reduce skewness of their distribution.

Parameters used to indicate the health-related physical fitness:

1. Resting heart rate: Resting heart rate will be measured following a seated ten-minute rest period. The heart rate will be detected by electronic device (Polar Electro, Finland).
2. Blood pressure: same as aforementioned
3. VO_2max_ (Maximum Oxygen Consumption): The concept of cardiovascular endurance is an individual’s aerobic capacity, and the most reliable and valid measure of aerobic capacity is VO_2max_ (Morrow, Jackson, Disch, & Mood, 2005). Bruce Protocol will be applied in the study. This test consists of several 3-minute stages, where the speed and grade are changed each stage, using the treadmill as a mode (ACSM, 2010). Table 1 illustrated the detail of the protocol:

Table 1: The Bruce Treadmill Protocol

| Stage | Minutes | Speed (mph) | Grade (%) |
| --- | --- | --- | --- |
| I | 1-3 | 1.7 | 10 |
| II | 4-6 | 2.5 | 12 |
| III | 7-9 | 3.4 | 14 |
| IV | 10-12 | 4.2 | 16 |
| V | 13-15 | 5.0 | 18 |
| VI | 16-18 | 5.5 | 20 |

1. Muscular endurance: The Canadian Standardized Test of Fitness - Push-up and Curl-up Tests will be conducted to measure the muscle endurance of upper-body muscles and the abdominal muscle groups, respectively. The test procedures for the measurements will be according to the descriptions from Canadian Society for Exercise Physiology (Canadian Society for Exercise Physiology, 2003, as cited in ACSM, 2010).
2. Flexibility: The modified back-saver sit-and-reach test (MBS test) will be used to test of trunk flexion. The MBS test is a comparative better test to measure the low back and hamstring flexibility than other protocols as its similarity of criterion-related validity in women but it has better criterion-related validity in men, more practical as it requires minimal preparation time and equipment. It also eliminates excessive posterior compression of the vertebral disk when performing a single leg reach (Hui & Yuen, 2000).
3. Anthropometric measurements: Body weight will be measured in light clothing and without shoes to the nearest 0.5 kg at the start and the end of the program. Height will be measured to the nearest 0.5 cm. Body mass index (BMI) will be calculated as weight in kilograms divided by height in meters squared.

Parameters used to indicate the effect on mental health:

1. Health related quality of life (HRQOL): the MOS 36-Item Short-Form Health Survey (SF-36) (Ware, Kosinski, & Gandek, 2000) includes one multi-item assessing each of eight health concepts: 1) physical functioning; 2) role limitations due to physical problems, 3) social functioning, 4) bodily pain, 5) general mental health, 6) role limitations due to emotional problems, 7) vitality, 8) general health perceptions. The internal consistency of the SF-36 ranged from .63 to .96 and the test–retest reliability ranged from .60 to .81 (Ware, Kosinski, & Gandek, 2000).
2. Perceived stress: Perceived Stress Scale (PSS) assesses the degree to which an individual appraises situations in his or her life as stressful (Cohen, Kamarck, & Mermelstein, 1983). It consists of 14 Likert-type items designed to assess how unpredictable, uncontrollable, and overloaded respondents perceived their lives to be. A lower score is indicative of lower perceived stress and score can range from 0 to 56. The PSS has been shown to be valid, with internal reliability as determined by Cronbach’s α of 0.902 (Smith et al., 2008).
3. Depression: The Centre for Epidemiologic Studies Depression Scale (CES-D) is a 20-item self-report scale to measure depressive symtomatology in the general population. The scale has high internal consistency with Cronbach’s α of 0.85 (Radloff, 1977). It yielded Cronbach’s α of 0.92 in the study of Mitchell et al. (2007).
4. Anxiety: State-Trait Anxiety Inventory (STAI) is a 40-item self-report scale to evaluate state and trait anxiety (Spielberger, 1983). Cronbach’s α for the state and trait subscales were 0.92 and 0.96, respectively, in the study of Mitchell, Mazzeo, Rausch, and Cooke (2007).
5. Self-esteem: Rosenberg Self-Esteem Scale (RSES) (Rosenberg, 1965) consists of 10-items, which examines the global self-esteem of individuals. The original scale is typically replaced with a Likert-type response format using a four-point scale anchored by 1 (Always Disagree) and 4 (Always Agree) (Sheasby, Barlow, Cullen, & Wright, 2000). The reproducibility of the scale is 93%, scalability of items is 73%, and scalability of individuals is 72 % (Rosenberg, 1965). Internal consistencies in the study of Elavsky and McAuley (2007) were good (α ranged from .89 to .92 for baseline and post-intervention assessments, respectively).

Physical activity level: The short (7 day) form of the International Physical Activity Questionnaire (IPAQ) (Craig et al., 2003) will be applied to investigate the subjects’ physical activity level. A study was carried out to examine the reliability and validity of IPAQ in 12 countries (Craig et al., 2003). The result showed that IPAQ had reasonable measurement properties for monitoring population levels of physical activity among 18 to 65 years old adults in the diverse sample settings. Spearman’s Rho clustered around 0.8 indicating reliable responses between repeat administrations for all versions of the IPAQ. The short IPAQ has been translated to Chinese and has demonstrated adequate reliability and validity for the measurement of total physical activity in a Chinese population (Macfarlane, Lee, Ho, Chan, & Chan, 2007).

The “Yoga and Health Questionnaire” was developed which consisted of the short form of IPAQ, RSES, PSS, CEDS, SF-36, and STAI in order to measurement the aforementioned parameters of each subjects. See Appendix A for the Chinese version of the questionnaire. Demographic data of each subject will be collected by the “Demographic Data Sheet” (See Appendix B). Subjects’ gender, age, education level, employment status, martial status, smoking and alcoholic intake will be asked.

Intervention

The intervention of the study will be a 12-week hatha yoga program. This program will consist of 12 weekly classes of one hour duration. All classes will be taught by the same instructor, who has three years of hatha yoga instructing experience. Sixty yogic poses and breathing commonly taught in fitness centres and private yoga studios will be instructed and practiced throughout the program (Kappmeier & Ambrosini, 2006; Kaminoff, 2007). Modification of some poses will be suggested if subjects have limitations in their flexibilities. Props such as mats and blocks will be used.

Study subject

Recruiting subjects:

Subjects will be screened for inclusion and exclusion criteria.

The inclusion criteria will be as follows:

1. A Chinese national and is able to communicate in Cantonese

2. Aged 18 or above

3. Is physically and mentally capable of practicing yoga safely

The exclusion criteria will be as follows:

1. Regular participation of (> 1/week) yoga for last 3 months or concurrent use of yoga, Qigong or meditation.
2. Pregnancy or breast feeding
3. Anticipate any planned life stressors (moving, divorce, changing job, etc.), shift work, or transcontinental travel during the intervention
4. Undergo any other concurrent nonpharmacological treatment of MetSyn
5. Chronic illnesses: cancer, kidney disease, cirrhosis, rheumatologic diseases, or chronic inflections
6. Current major psychiatric illnesses, cognitive impairment, or substance abuse
7. Concurrent enrollment in any other studies, experimental therapies, or blinded treatments.

If there would be change of subjects’ health status such as major psychiatric illnesses, chronic illnesses (e.g. cancer, kidney disease, cirrhosis, rheumatologic diseases), chronic inflections, cognitive impairment, substance abuse or pregnancy would be found during the participation of the study, the subjects are required to inform the research assistant and be withdrawn from the study.

Grouping:

The number of revised ATP III criteria that each subject meets will be assessed during the baseline assessment, and subjects will be assigned into MetSyn group (for those who meet three or more out of five ATP III criteria) and non-MetSyn group (for those who meet less than three out of five ATP III criteria) based on the number of the criteria they met.

Subjects in both MetSyn and non-MetSyn groups will be assigned into the yoga group or waitlist control group. Subjects assigned to the waitlist control group will be asked to complete the same measures as their yoga group counterparts but received no treatment. To ensure acceptance of the protocol, the waitlist control subjects will be offered a free 12-week hatha yoga program upon completion of the trial.

Sample size estimation:

Due to lacking of local research experience in running hatha yoga intervention within the community, the estimation of attrition rate will be made by referring to three overseas studies with similar study setting which illustrated a 22.0% attrition for a 12-week hatha yoga program (Moadel et al., 2007), a 15.3% attrition for a 10-week hatha yoga program (Smith, Hancock, Blake-Mortimer, & Eckert, 2007), and a 8.33% attrition for another 10-week program (Booth-LaForce, Thurston, & Taylor, 2007). Because the study intervention would last for 12 weeks, a 22.0 % attrition rate will be estimated.

Based on an earlier study (West, Otte, Geher, Johnson, & Mohr, 2004) and assuming 22% attrition rate, a sample size of 46 subjects per group is required to obtain an 80% power for detection of a difference of 0.08 g/dl in salivary cortisol level between the Yoga group (intervention group) and the control group when performing a two-tailed test at α=0.05. The calculation was illustrated as followed:

n = 2σ^2^ [z(α/2) + z(β)]^2^ where σ = 0.121 and Δ=0.08

Δ^2^

= 2*0.121^2^ [1.96 +0.842)]^2^

0.08^2^

= 35.9 (= 36 subjects per group when round up)

= 46 for assuming attrition rate at 22%.

The number of subjects required for the project will be estimated according to following groupings:

Pilot Study group: 20; Main study: 184 in which

MetSyn group: 46 x 2 = 92 (yoga group and control group) and

Non-MetSyn: 46 x 2 = 92 (yoga group and control group).

Thus, the sample size = 20 + 92 + 92 = 204

Procedures to be applied to subjects

Written consent (see Appendix C) and PAR-Q (see Appendix D) will be signed by the subjects after the background of the study and the procedures to be applied to the subjects have been explained to the subjects. The subjects will be screened and classified to MetSyn group or Non-MetSyn group according to the revised ATP III criteria aforementioned, and assigned to either yoga group or waitlist control group. All subjects will attend one baseline assessment session and one post-intervention assessment session in the study. On each assessment session, blood sample will be taken from each subject. The physical fitness tests aforementioned will conducted and the “Physical Fitness Assessment Record” (see Appendix E) will be completed. Each subject will be interviewed by the research assistant to complete the Yoga and Health Questionnaire and Demographic Questionnaire.

During the 12-week intervention, subjects in the yoga group will be asked to attend the yoga program described in the intervention part. Hatha Yoga Class Handout (see Appendix F), presented the picture of each pose taught in each lesson, will be given to the subjects in the first 10 lessons. They will be strongly encouraged to attend all the 12 lessons and practice at home every day. The subjects will receive “Yoga Self Practice Weekly Record Sheet” (see Appendix G) after each lesson and be asked to record if they practice yoga and for how long. The completed sheet will be collected in the lesson of the following week, except the sheet recorded the 12^th^ week will be collected in post intervention assessment session. Class attendance also will be recorded. Salivary cortisol samples will be taken from each subject pre and post the first and the last yoga lesson. Subjects in the waitlist control group will be asked to maintain their routine activities and not begin any exercise, yoga or mind-body program during the course of the study. Their salivary cortisol samples will be taken at the same timeslot as those counterparts in the yoga group.

Pilot Study

A pilot study will be carried out in various ways to test the feasibility of the data collection method and the study intervention, and to guarantee that the main study will be implemented smoothly and successfully according to what have proposed.

The feasibility of the data collection method included the instruments used and the process. To test if the data collection instruments are feasible, subjects’ responses towards the understanding of the questionnaires and the ease of the physiological tests will be examined. The time needed to complete data collection instrument will be measured.

The feasibility of the study intervention will be examined in terms of intensity and arrangement. To pilot the intervention intensity, the subject will invite to join a 12-week hatha yoga program with the same intensity as the main study. After the completion of pilot program, the subjects will be asked if they would encounter any difficulties and experience any discomfort during the pilot program. To pilot the arrangement, the instructor, time and venue will be arranged exactly the same as in the main study. Any technical problems will spot out and solve as soon as possible. The pilot in the above ways would serve to obtain important information regarding to the feasibility of the main study and provide initial evidence on the effectiveness of the hatha yoga program.

Data Analysis

The statistical analysis will be conducted using SPSS 16.0 software (SPSS Inc, Chicago, Illinois). Sample description will be described; baseline characteristics, retention and program adherence, and all other outcome measures will be analyzed and reported. Paired t test and independent t test will be used to analyze continuous variables. Mann-Whitney test will be used to analyze continuous variables which did not have normal distribution. Chi square test will be employed to compare categorical variables. Statistical significance will be inferred by a two-tailed p value of .05 or less.

**Reference:**

American College of Sports Medicine (ACSM). (2010). *ACSM's guidelines for exercise testing and prescription* (8^th^ ed.). Philadelphia: Wolters Kluwer Health/Lippincott Williams & Wilkins

Beddoe, A. E., Yang, C. P. P., Kennedy, H. P., Weiss, S. J., & Lee, K. A. (2009). The effects of mindfulness-based yoga during pregnancy on maternal psychological and physical distress.  *Journal of Obstetric, Gynecologic, and Neonatal Nursing: JOGNN*, *38* (3), 310-319.

Björntorp, P., & Rosmond, R. (1999). Hypothalamic origin of the Metabolic Syndrome X. *Annals of the New York Academy of Sciences*, *892*, 297-307

Björntorp, P., & Rosmond, R. (2000). Neuoendocrine abnormalities in visceral obesity. *International Journal of Obesity*, *24* (Supp 2), 80-85*.*

Booth-LaForce, C., Thurston, R. C., & Taylor, M. R. (2007). A pilot study of a hatha yoga treatment for menopausal symptoms. *Maturitas*, *57*, 286-295.

Bosch, P. R., Traustadottir, T., Howard, P., & Matt, K. S. (2009). Functional and physiological effects of yoga in women with rheumatoid arthritis: a pilot study. *Alternative Therapies in Health and Medicine*, *15* (4), 24-31.

Bukowski, E. L., Conway, A., Glentz, L. A., Kurland, K., & Galantino, M. L. (2007). The effect of iyengar yoga and strengthening exercises for people living with osteoarthritis of the knee: a case series. *International Quarterly of Community Health Education*, *26* (3), 287-305.

Chen, K. M., Chen, M. H., Hong, S. H., Chao, H. C., Lin, H. S., & Li, C. H. (2008). Physical fitness of older adults in senior activity centres after 24-week silver yoga exercises. *Journal of Clinical Nursing*, *17*, 2634-2646.

Chen, K. M., Tseng, W. S., Ting, L. F., & Huang, G. F. (2007). Development and evaluation of a yoga exercise programme for older adults. *Journal of Advanced Nursing*, *57* (4), 432-441.

Chrousos, G. P. (2000). The role of stress and the hypothalamic-pituitary-adrenal axis in the pathogenesis of the metabolic syndrome: neuro-endocrine and target tissue-related causes. *International Journal of Obesity*, *24* (Supp 2), 50-55.

Cohen, S., Kamarck, T., & Mermelstein, R. (1983). A global measure of perceived stress. *Journal of Health and Social Behaviour*, *24*, 385-396.

Craig, C. L., Marshall, A. L. , Sjostrom, M., Bauman, A. E. , Booth, M. L. , Ainsworth, B. E., et al. International physical activity questionnaire: 12-country reliability and validity. (2003). Medicine and science in sports and exercise, 35(8), 1381-1395.

Culos-Reed, S. N., Carlson, L. E., Daroux, L. M., & Hately-Aldous, S. (2006). A pilot study of yoga for breast cancer survivors: physical and psychological benefits. *Psycho-Oncology*, *15*, 891-897.

DiStasio, S. A. (2008). Integrating yoga into cancer care. *Clinical Journal of Oncology Nursing*, *12* (1), 125-130.

Donesky-Curenco, D., Nguyen, H. Q., Paul, S., & Carrier-Kohlman, V. (2009). Yoga therapy decreases dyspnea-related distress and improves functional performance in people with chronic obstructive pulmonary disease: a pilot study. *The Journal of Alternative and Complementary Medicine*, *15* (3), *225-234*.

Elavsky, S., & McAuley, E. (2007). Exercise and self-esteem in menopausal women: a randomized controlled trial involving walking and yoga. *American Journal of Health Promotion*, *22* (2), 83-92.

Fowler, S. B., Moussouttas, M., & Mancini, B. (2005). Metabolic Syndrome: Contributing Factors and Treatment Strategies. *Journal of Neuroscience Nursing*, *37* (4), 220-223.

Franks, P. W., Ekelund, U., Brage, S., Wong, M.Y., & Wareham, N. J. (2004). Does the association of habitual physical activity with the metabolic syndrome differ by level of cardiorespiratory fitness? *Diabetes Care*, *27* (5), 1187-1193.

Galentino, M. L., Bzdewka, T. M., Eissler-Russo, J. L., Holbrook, M. L., Mogek, E. P., & Farrar, J. T. (2004). The impact of modified hatha yoga on chronic low back pain: a pilot study. *Alternative Therapies in Health and Medicine*, *10* (2), 56-59.

Granath, J., Ingvarsson, S., von Thiele, U., & Lundberg, U. (2006). Stress management: a randomized study of cognitive behavioural therapy and yoga. *Cognitive Behaviour Therapy*, *35* (1), 3-10.

Groessl, E. J., Weingart, K. R., Aschbacher, K., Pada, L., & Naxi, S. (2008). Yoga for veterans with chronic low-back pain. *The Journal of Alternative and Complementary Medicine*, *14* (9), 1123-1129.

Grundy, S. M., Cleeman, J. I., Daniels, S. R., Donato, K. A., Eckel, R. H., Franklin, B. A., et al. (2005). Diagnosis and management of the metabolic syndrome. An American Heart Association/National Heart, Lung, and Blood Institute scientific statement. *Circulation*, *112*, 2735-2752.

Hui, S. C., & Yuen, P. Y. (2000). Validity of the Modified Back Saver Sit-and-Reach Test: A Comparison with Other Protocols. *Medicine and Science in Sports and Exercise*, *32* (9), 1655-1659.

Hwang, L. C., Bai, C. H., Chen, C. J., & Chien, K. L. (2007). Gender difference on the development of metabolic syndrome: a population-based study in Taiwan. *European Journal of Epidemiology*, *22*, 899-906.

Innes, K. E., Selfe, T. K., & Taylor, A. G. (2008). Menopause, the metabolic syndrome, and mind-body therapies. *Menopause*, *15* (5), 1005-1013.

Innes, K. E., Vincent, H. K., & Taylor, A. G. (2007). Chronic stress and insulin resistance-related indices of cardiovascular disease risk, part 2: a potential role for mind-body therapies. *Alternative Therapies*, *13* (5), 44-51.

Johnson, J. L., Slentz, C. A., Houmard, J. A., Samsa, G. P., Duscha, B. D., Aiken, L. B., et al. (2007). Exercise training amount and intensity effects on metabolic syndrome (from studies of a targeted risk reduction intervention through defined exercise). *The American Journal of Cardiology*, *100*, 1759-1766.

Kaminoff, L. (2007). *Yoga Anatomy*. US: Human Kinetics.

Kappmeier, K. L., & Ambrosini, D. M. (2006). *Instructing Hatha Yoga*. Champaign, IL: Human Kinetics.

Khalsa, S. B. S. (2004). Treatment of chronic insomnia with yoga: a preliminary study with sleep-wake diaries. *Applied Psychophysiology and Biofeedback*, *29* (4), 269-278.

Khattab, K., Khattab, A. A., Ortak, J., Richardt, G., & Bonnermeier, H. (2007). Iyengar yoga increases cardiac parasympathetic nervous modulation among healthy yoga practitioners. *Evidence-based Complementary and Alternative Medicine*, *4* (4), 511-517.

Kirschbaum, C., & Hellhammer, D.H. (1989). Salivary cortisol in psychobiological research: an overview. *Neuropsychobiological*, *22*, 150-169.

Kraemer, J. M., & Marquez, D. X. (2009). Psychological correlates and outcomes of yoga or walking among older adults. *The Journal of Psychology*, *143* (4), 390-404.

Kyrou, I., Chrousos, G. P., & Tsigos, C. (2006). Stress, visceral obesity, and metabolic complications. *Annals New York Academy of Sciences*, *1083*, 77-110.

Lavey, R., Sherman, T., Mueser, K. T., Osborne, D. D., Currier, M., & Wolfe, R. (2005). The Effects of Yoga on Mood in Psychiatric Inpatients. *Psychiatric Rehabilitation Journal*, *28* (4), 399-402.

Lee, M. S., Kim, J., Ha, J. Y., Boddy, K., & Ernst, E. (2009). Yoga for menopausal symptoms: a systematic review. *The Journal of the North American Menopause Society*, *16* (3), 602-608.

Levine, M. (2000). *The positive psychology of Buddhism and yoga: path to a mature happiness with a special application to handle anger*. Mahwah, N.J.: Lawrence Erlbaum Publishers

Macfarlane, D. J., Lee, C. C. Y., Ho, E. Y. K., Chan, K. L., & Chan, D. T. S. (2007). Reliability and validity of the Chinese version of IPAQ (short, last 7 days). *Journal of Science and Medicine in Sport*, *10*, 45–51.

Mclver, S., O’Halloran, P., & McGartland, M. (2009). Yoga as a treatment for binge eating disorder: a preliminary study. *Complementary Therapies in Medicine*, *17*, 196-202.

Mendelson, S. D. (2008). *Metabolic Syndrome and Psychiatric Illness: interactions, pathophysiology, assessment and treatment*. Amsterdam; Boston: Elsevier/ Academic Press.

Michalsen, A., Grossman, P., Acil, A., Langhorst, J., Lüdtke, R., Esch, T., Stefano, G. B., & Dobos, G. J. (2005). Rapid stress reduction and anxiolysis among distressed women as a consequence of a three-month intensive yoga program. *Medical Science Monitor*, *11* (12), 555-561.

Mitchell, K. S., Mazzeo, S. E., Rausch, S. M., & Cooke, K. L. (2007). Innovative interventions for disordered eating: evaluating dissonance-based and yoga intervention. *International Journal of Eating Disorders*, *40* (2), 120 - 128.

Mittal, S. (2008). *The Metabolic Syndrome in Clinical Practice*. London: Springer.

Moadel, A. B., Shah, C., Wylie-Rosett, J., Harris, M. S., Patel, S. R., Hall, C.B., et al. (2007). Randomized controlled trial of yoga among a multiethnic sample of breast cancer patients: effects on quality of life. *Journal of Clinical Oncology*, *25* (28), 4387-4395.

Morley, J. E., & Sinclair, A. (2009). The metabolic syndrome in older persons: a loosely defined constellation of symptoms or a distinct entity? *Age and Ageing*, *38*, 494-497.

Morrow, J. R. Jr., Jackson, A. W., Disch, J. G., & Mood, D. P. (2005). *Measurement and Evaluation in Human Performance* (3^rd^ ed.). US: Human Kinetics.

Mourya, M., Mahajan, A. S., Singh, N. P., & Jain. A. K. (2009). Effect of slow- and fast-breathing exercises on autonomic functions in patients with essential hypertenstion. *The Journal of Alternative and Complementary Medicine*, *15* (7), 711-717.

Pickering, T. G., Hall, J. E., Appel, L. J., Falkner, B. E., Graves, J., Hill, M. N., et al. (2005). AHA scientific statement: Recommendations for blood pressure measurement in humans and experimental animals. *Hypertension*, *45*, 142-161.

Pilkington, K., Kirkwood, G., Rampes, H., & Richardson, J. (2005). Yoga for depression: the research evidence. *Journal of Affective Disorder*, *25* (89), 13-24.

Oken, B. S., Zajdel, D., Kishiyama, S., Flegal, K., Dehen, C., Haas, M., et al. (2006). Randomized, controlled, six-month trial of yoga in healthy seniors: effects on cognition and quality of life. *Alternative Therapies in Health and Medicine*, *12* (1), 40-47.

Radloff, L. S. (1977). The CES-D scale: A self-report depression scale for research in the general population. *Applied Psychological Measurement*, *1*, 385-401.

Reaven, G. M. (1988) Banting lecture 1988: role of insulin resistance in human disease. *Diabetes*, *37*, 1595-1607.

Rennie, K. L., McCarthy, N., Yazdgerdi, S., Marmot, M., & Brunner, E. (2003). Association of the metabolic syndrome with both vigorous and moderate physical activity. *International Journal of Epidemiology*, *32* (4), 600-606.

Riley, D. (2004). Hatha yoga and the treatment of illness. *Alternative Therapies in Heath and Medicine*, *10* (2), 20-21.

Ripoll, E., & Mahowald, D. (2002). Hatha yoga therapy management of urologic disorders. *World Journal of Urology*, *20*, 306-309.

Roberts-Grey, G. (2008). On the pulse: preventing Syndrome X. *American Fitness*, *26* (4), 20

Rosenberg, M. (1965). Society and the Adolescent Self-image. Princeton, N.J.: Princeton University Press.

Rosmond, R. (2005). Role of stress in the pathogenesis of the metabolic syndrome. *Psychoneuroendocrinology*, *30*, 1-10.

Saper, R. B., Eisenberg, D. M., Davis, R. B., Culpepper, L., & Phillips, R. S. (2004). Prevalence and patterns of adult yoga use in the United States: results of a national survey. *Alternative Therapies in Health and Medicine*, *10* (2), 44-49.

Satyapriya, M., Nagendra, H. R., Nagarathna, R., & Padmalatha, V. (2009). Effect of integrated yoga on stress and heart rate variability in pregnant women. *International Journal of Gynecology and Obstetrics*, *104*, 218-222.

Sheasby, J. E., Barlow, J. H., Cullen, L. A., & Wright, C. C. (2000). Pyschometric properties of the Rosenberg Self-Esteem Scale among people with arthritis. *Psychological Reports*, *86*, 1139-1146.

Smith, B. W., Shelley, B. M., Dalen., J., Wiggins, K., Tooley E., & Bernard, J. (2008). A pilot study comparing the effects of mindfulness-based and cognitive-behavioral stress reduction. *The Journal of Alternative and Complementary Medicine*, *14* (3), 251-258.

Smith, C., Hancock, H., Blake-Mortimer, J., & Eckert, K. (2007). A randomised comparative trial of yoga and relaxation to reduce stress and anxiety. *Complementary Therapies in Medicine*, *15*, 77-83.

Spielberger, C. T. (1983) *Manual for the Stait-Trait Anxiety Inventory (form Y)*. Palo Alto: Consulting Psychologists Press

Sullivan, V. K. (2006). Prevention and treatment of the metabolic syndrome with lifestyle intervention: where do we start? *Journal of the American Dietetic Association*, *106* (5), 668-671.

Tan, C. E., Chew, S. K., Ma, S., Tai, E. S., & Wai, D. (2004). Can we apply the National Cholesterol Education Program Adult Treatment Panel definition of the metabolic syndrome to Asians? *Diabetes Care*, *27* (5), 1182-1186.

Telles, S., Joshi, M., Dashi, M., Raghuraj, P., Naveen, K. V., & Nagendra, H. R. (2004). An evaluation of the ability to voluntarily reduce the heart rate after a month of yoga practice. *Integrative Physiological & Behavioral Science*, *39* (2), 119-125.

Tekur, P., Singhow, C., Nagendra, H. R., & Raghuram, N. (2008). Effect of short-term intensive yoga program on pain, functional disability, and spinal flexibility in chronic low back pain: a randomized control study. *The Journal of Alternative and Complementary Medicine*, *14* (6), 637-644.

Thomas, G. N., Ho, S. Y., Janus, E. D., Lam, K. S. L., Hedley, A. J., & Lam, T. H. (2005). The US National Cholesterol Education Programme Adult Treatment Panel III (NCEP ATP III) prevalence of the metabolic syndrome in a Chinese population. *Diabetes Research and Clinical Practice*, *67*, 251-257.

Tong, P. C., Kong, A. P., So, W. Y., Yang, X., Ho, C. S., Ma, R. C., et al (2007). The usefulness of the International Diabetes Federation and the National Cholesterol Education Program's Adult Treatment Panel III definitions of the metabolic syndrome in predicting coronary heart disease in subjects with type 2 diabetes. *Diabetes Care*, *30* (5), 1206-1211.

Tsigos, C., & Chrousos, G. P. (2002). Hypothalamic-pituitary-adrenal axis, neuroendocrine factors and stress. *Journal of Psychosomatic Research*, *53*, 865-871.

Vadiraja, H. S., Raghavendra, R. M., Nagarathna, R., Nagendra, H. R., Rehka, M., Vanitha, N., et al. (2009). Effects of a yoga program on cortisol rhythm and mood states in early breast cancer patients undergoing adjuvant radiotherapy: a randomized controlled trial. *Integrative Cancer Therapies*, *8* (1), 37-46.

Vera, F. M., Manzaneque, J. M., Maldonado, E. F., Carranque, G. A., Rodriguez, F. M., Blanca, M. J., & Morell, M. (2009). Subjective Sleep Quality and hormonal modulation in long-term yoga practitioners. *Biological Psychology*, *81*, 164-168.

Visweswaraiah, N. K., & Telles, S. (2004). Randomized trial of yoga as a complementary therapy for pulmonary tuberculosis. *Respirology*, *9*, 96-101.

Ware, J. E., Kosinski, M., & Gandek, B. (2000). *SF-36 health survey: manual & interpretation guide*. Lincon, RI: QualityMetric Incorporated.

West, J., Otte, C., Geher, K. Johnson, J., & Mohr, D. C. (2004). Effects of hatha yoga and African dance on perceived stress, affect, and salivary cortisol. *Annals of behavioral medicine*, *28* (2), 114-118.

Wijndaele, K. Duvigneaud, N., Matton, L., Duquet, W., Delcluse, C., Thomis, M., et al. (2009). Sedentary behaviour, physical activity and a continuous metabolic syndrome risk score in adults. European Journal of Clinical Nutrition, 63, 421-429.

Woolery, A., Myers, H., Sternlieb, B., & Zeltzer, L. (2004). A yoga intervention for young adults with elevated symptoms of depression. *Alternative Therapies in Health and Medicine*, *10* (2), 60-63.
